# Distinguishing appropriate from inappropriate conditions on research participation

**DOI:** 10.1111/bioe.13092

**Published:** 2022-10-21

**Authors:** Robert Steel, David Wendler

**Affiliations:** ^1^ Department of Philosophy University of Nebraska at Omaha Omaha Nebraska USA; ^2^ Department of Bioethics NIH Clinical Center Bethesda Maryland USA

**Keywords:** genetic research, human subjects research, moral theory, regulatory issues, research ethics

## Abstract

Individuals do not have a right to participate in clinical trials. But, they do have a right against being denied participation for inappropriate reasons. Despite the widespread endorsement of these two claims, there has been little discussion regarding which conditions for participation in clinical trials are appropriate and which are inappropriate. The present manuscript attempts to address this gap in the literature. We first describe and then argue against the claim that conditions on enrollment or continued participation are appropriate only when they are needed to answer the scientific question(s) posed by the trial. We then offer an alternative view according to which the appropriateness of conditions depends on whether they help to satisfy the ethical requirements of clinical research. Because these requirements include social value, the present view implies that promoting social value is an acceptable reason to impose conditions on research participation. With this in mind, we explain why it is not coercive to require potential participants to accept conditions on enrollment that promote a trial's social value, even when the participants find those conditions unwelcome. We conclude by evaluating the present proposal's implications for the common practice of requiring participants to agree to the possible use of their leftover biospecimens in a broad range of future research. We argue, contra current regulatory policy, that this practice can be acceptable even when the present trial offers participants the prospect of clinical benefit and the samples are being reserved for future research that is unrelated to the present trial.

## 1 INTRODUCTION

It is commonly said that individuals do not have a right to participate in clinical research.^1^ At the same time, individuals do have a right against being denied participation for inappropriate reasons.^2^ But what makes a reason appropriate or inappropriate? Despite the importance of this question, there have been, to our knowledge, few attempts to address it, and no generally accepted answer has been identified in the literature. In this paper, we describe and endorse an approach to developing one.

In doing so, we will rely on the notion of *conditions* for participation in clinical research. As we use the term, conditions on participation are not medical conditions relevant to the study in question (e.g., cancer). Instead, they are requirements a participant must satisfy if they are to participate in a given study. Inclusion and exclusion criteria are explicitly enumerated conditions, as participants must meet inclusion criteria, and not meet exclusion criteria, in order to enroll. But the notion of a condition on participation is broad. Conditions need not be explicitly stated and may apply throughout participation, not just at enrollment. For instance, a common though rarely explicitly stated condition on research participation is that participants do not behave in ways that are violent or abusive toward research staff. If a participant leaves 26 consecutive threatening voicemails on a researcher's phone, they will be dropped from the trial. That is but one among many more‐ and‐ less‐ explicit expectations and requirements routinely applied to research participants. All such requirements are, in our sense, conditions for participation in research.

Having explained the notion of a condition on participation, we can now express the question we started with as follows: What conditions on participation are appropriate, and which ones are inappropriate? Few would object to the use of scientifically justifiable inclusion and exclusion criteria or to dropping participants who behave threateningly. Such conditions are obviously appropriate. On the other hand, imagine that researchers demand that participants in a trial come to the researchers' homes and wash their cars on the weekends and that anyone who refuses is dropped from the trial. That condition is obviously inappropriate. In addition to the easy cases, there are conditions whose status is less immediately clear. Consider, for instance, a question that arises in the context of many trials: can it be appropriate for researchers to require, as a condition of participation, that participants agree to allow their leftover biosamples to be stored and made available for future research, including research that is not related to the present trial?

A version of this question has recently been addressed by OHRP, the U.S. Office of Humans Research Protections. Specifically, OHRP issued a determination that, in studies that offer participants a prospect of clinical (or “direct”) benefit, participants cannot be required to agree to allow their leftover biosamples to be stored and made available for future, potentially unrelated research because such a demand is coercive.^3^ OHRP's determinations are binding and institutions must change their policies and practices to bring them into compliance.^4^ As we later discuss, the NIH intramural IRB has done so in response to this determination. This determination is hence of great practical interest. And assessing whether it was correct requires a theory of which conditions for participation in clinical research are appropriate and which conditions are inappropriate.

One view, which supports OHRP's position, is what we call the “scientific necessity” view.^5^ In this view, conditions on enrollment in trials that offer the prospect of clinical benefit are appropriate only when they are needed to answer the scientific question(s) posed by the study. This view gains support from the fact that potential participants may feel forced to accept mandated conditions and seems especially plausible when applied to studies that offer participants the prospect of direct benefit. In this view, the only acceptable justification for putting potential participants in the position of having to choose between complying with an unwanted condition or forgoing what may be their only chance to access potentially beneficial treatment is that the condition is necessary to answer the scientific question(s) posed by the study.

The scientific necessity view makes sense to the extent that one understands clinical trials purely as scientific activities designed to collect specified information for the purposes of addressing questions that are relevant to improving health and well‐being. While this characterization is accurate, it is also incomplete. Clinical trials are joint activities that are part of the practice of clinical research, which is a broad and complex social institution that involves a range of stakeholders. As such, clinical trials raise a number of distinct and sometimes conflicting concerns and are governed by a range of ethical norms.

This broader understanding of clinical research suggests a different account of appropriate conditions for participation. Specifically, it suggests that whether a condition is appropriate depends on whether it on balance furthers the full range of stakeholder interests and governing norms. If it does, it is appropriate, and it is, therefore, acceptable to offer potential participants the choice of either agreeing to that condition or declining to enroll. This can be true even for unwelcome conditions, and even in the context of trials that offer participants the prospect of direct benefit. In this paper, we will both argue for and apply this broader account.

We do so in three parts. In the first part, we argue against the “scientific necessity” view by documenting commonplace conditions that researchers place on research enrollment which are plainly acceptable, despite being unnecessary to answering the scientific question(s) posed by the studies in question. In the second part, we describe our alternative view and explain why the conditions it supports, even when unnecessary to answer the study question(s) and potentially unwelcome to participants, can nonetheless be appropriate. In the third part, we apply our view to the mandatory retention of leftover biosamples for unspecified future research, using OHRP's position as our foil. Our analysis suggests that placing this condition on research participation is not only typically ethically permissible but, perhaps surprisingly, may be ethically required in many cases. Our conclusions thus support a change to current U.S. regulatory policy. Further research is needed to determine the implications for other conditions on research enrollment.

### 1.1 Are mandatory conditions ever appropriate?

Initially, one might think that placing any conditions at all on potential participants' decision whether to enroll in a clinical trial is inappropriate. After all, one of the ethical requirements of clinical research is to obtain participants' *voluntary* consent. Consider an individual who is deciding whether to enroll in a phase 3 study that is evaluating a potential new treatment that has shown initial promise for treating bladder cancer. Assuming the individual is eligible (e.g., she has bladder cancer), placing conditions on her enrollment may seem like inappropriate obstacles to her accessing a potentially beneficial medical treatment. It is saying, in effect: We will permit you to access a potential new treatment for your significant medical condition, but only if you agree to do what we require.

The claim that placing any mandatory conditions on clinical trials is incompatible with voluntary consent has an initial appeal. Yet essentially all voluntary agreements come with some conditions. Consider standard clinical care. Patients with cancer are not offered access to medical treatment free from any and all conditions. They are required to show up at specific places and times. They must agree to necessary tests and scans, and measures to protect the staff (e.g., wearing a mask). Importantly, these conditions are appropriate even when they are not strictly necessary for the patient to access the treatment, and even when they impose burdens the patient would prefer to avoid.

Research is no different. It is uncontroversial that potential trial participants can also be required to show up at specific places and times, and agree to necessary tests and scans, and measures to protect the staff (again including wearing a mask). They can also be required to abide by research‐specific conditions, such as the requirement to track and report adverse events honestly. When participants seek to enroll, they are required to agree to all these conditions and many others besides, and those who refuse are denied enrollment. Those who initially agree, but then fail to honor that agreement may be dropped from the trial. This is true even for trials that offer a prospect of medical benefit and even when the individual has no treatment options outside of research. This is a critical point, as it suggests that whatever might be wrong with placing certain conditions on research participation, it cannot be that the imposition of any conditions whatsoever is inconsistent with obtaining voluntary consent.

One might respond to these observations by conceding that the requirements we've cited so far are appropriate, but still go on to object that they are not best understood as being “conditions on participation” at all. This, the objection continues, is because they are not separate requirements that are *added* to trial participation, but are instead just explanations of what it is to participate in a given trial. If this is right, it suggests that one could accept these requirements as appropriate, while still maintaining that the imposition of any genuine conditions nonetheless violates the requirement to obtain participants' voluntary consent.^6^

To unpack this possibility, consider an extreme example. Imagine a person seeks to enroll in a clinical trial of a new drug but rejects *all* conditions researchers specify. That person is not interested in showing up to the clinic at all, let alone at any specified time. They will not submit to having blood drawn or allow their data to be collected. In fact, they even reject the administration of the study drug itself. In every respect, they insist that they intend to carry on as in their normal life. Nonetheless, they request that they be included in the trial. This would be perplexing. After all, one might think that what it is to be in a clinical trial of an experimental drug is to take that drug under certain controlled conditions, while being scientifically monitored, for the purpose of generating generalizable knowledge. If so, there is simply no such thing as “being in a trial” while sitting at home, doing none of these things.

This thought experiment suggests the possibility of dividing conditions on research enrollment into two categories. Some conditions simply express the constitutive features of the trial. These are not *extra* hoops participants must jump through in order to participate in the trial and access the experimental intervention—rather, they just explain what participation in the trial involves. For this reason, they are not in tension with the requirement to obtain voluntary consent and are therefore acceptable. But any conditions which go beyond the constitutive features of the trial, however, *would* be extra hoops, and requiring participants to jump through them would be inconsistent with voluntary consent. But using this approach to assess which conditions on research enrollment are acceptable requires a substantive view of what the constitutive features of a trial are. Specifically: how do we distinguish between constitutive features and extra hoops?

In a purely conventional sense, a trial just consists in whatever is described in the research protocol. But, if that were the sense being employed, then *anything* could be made a constitutive feature of a trial, and hence appropriately required of participants, simply by writing it into the protocol. Researchers could write “participants must wash researchers' cars” into the protocol, in which case washing researchers' cars would just be “part of what it is to participate in the trial.” Given that this purely conventional understanding would trivialize the view that only constitutive trial features can be legitimately imposed, we put it to the side.

A more plausible account of the constitutive features of clinical trials appeals to their scientific unity as a collection of interventions and procedures designed to answer a scientific question or questions. Such an account might say, for instance, that if a trial is attempting to determine whether a given experimental agent is safe and effective for treating bladder cancer, a biopsy that is needed to answer that question is a constitutive element of the trial. In contrast, washing a researcher's car has no scientific connection to systematically assessing the experimental agent's safety and efficacy—and consequently cannot be “part of what it is to participate” in the trial regardless of whether researchers were to (inappropriately) write it into the research protocol. This understanding of the constitutive features of a trial suggests that researchers may impose conditions on trial participation *only if* their inclusion is necessary to conduct the trial, where “the trial” is understood as a scientifically unified, systematic attempt to answer a specified question or questions. We assess this view in the next section.

### 1.2 Appropriate conditions as scientifically necessary or useful conditions

The view under consideration maintains that conditions which are necessary to answer the scientific question(s) posed by the trial are appropriate, whereas conditions that are not scientifically necessary are not appropriate.^7^ The strictest version of this view would specify that only conditions which are necessary for answering the *primary* question posed by the study are appropriate. However, almost all clinical trials that assess the efficacy of experimental interventions include secondary and tertiary scientific aims. For example, they might also assess potential side effects of the intervention and whether the intervention interacts with other medications taken by individuals with the condition under study. Achieving these aims typically greatly enhances a study's scientific and social value at relatively little additional cost and risk. To accommodate this practice, the scientific necessity view might be understood less strictly as permitting conditions that are necessary to answering any of the study's scientific questions, not just the primary one. Yet even this relaxed version still faces an objection that applies to all versions of the scientific necessity view.

Strictly speaking, researchers could answer the scientific questions posed by a study without mandating any conditions on enrollment at all. They could provide participants with the experimental intervention and then invite, rather than require, them to undergo the tests needed to assess its safety and efficacy. Of course, if some participants decline the tests, the value of the study results would be diminished. And, if many participants decline, investigators would have to increase enrollment. These trials would be more expensive and take longer, reducing the number of patients who benefit from the successful results of clinical trials. More participants would be exposed to potentially unsafe interventions, and a greater number of trials would fail to produce meaningful results. So, while it is *possible* to make all tests optional while still ultimately answering the study questions, doing so would massively increase the costs, burdens, and risks of conducting clinical trials, while also decreasing their benefits. In sum, the possibility of making the tests needed to assess safety and efficacy optional reveals that it is not scientifically necessary to mandate them as conditions on trial enrollment. The imprudence of doing so reveals that conditions need not be scientifically necessary to be legitimately imposed.

In response, one might concede that scientific necessity is an indefensible criterion but go on to argue that conditions on research enrollment are appropriate only when they are scientifically *useful*, even if not strictly necessary, for answering study questions. A scientific usefulness standard could explain why tests and scans can legitimately be required, even if a scientific necessity standard cannot. But this account, while an improvement, still seems too narrow. Permitting conditions on research enrollment only when they are scientifically useful would preclude researchers from mandating conditions that are important for realizing other legitimate goals beyond answering the scientific questions posed by the study.

Consider the almost universally endorsed goal of minimizing research risks. Researchers mandate that participants not wear their metal wedding bands when undergoing MRI scans, even when the participants would prefer not to remove them, and even when wearing them will not interfere with collecting valid data. Similarly, studies that assess psychoactive interventions standardly require participants to remain in the clinic after research testing is completed, until the effects of the intervention have subsided, before driving home. These widely‐endorsed requirements are not needed, or even useful for the purposes of answering the scientific questions posed by studies that include them. Instead, they are useful for reducing the risks of the trial to participants and others.

Importantly, minimizing risks is not the only legitimate goal of research, outside of answering the scientific question(s) posed by research studies. Consider another example: clinical trials standardly require participants to come to specified study sites on specified days of the week, rather than permitting them to be seen at whichever hospitals, and on whichever days they prefer. In some cases, these decisions are justified on the grounds that they are scientifically useful. For example, researchers might limit a study to certain sites because the expertise or equipment necessary for administering the experimental intervention is only available there. Similarly, conducting study procedures at specific times of the day or the month may be important to control for circadian rhythms or menstrual cycles. But, in other cases, these conditions are not scientifically necessary, or even useful.

Of course, study procedures must be conducted on some day, at some time, and in some place. That is clearly a constitutive feature of clinical trials. But, for many trials, there is no scientific reason for participants to present at a particular time. The consent to those trials could specify that the choice of time will be left to individual participants, allowing them to build a bespoke schedule. Some might present at 3 p.m., others at 3 a.m., depending on their preferences. But rather than take this approach, clinical trials almost always specify times and dates. Even when it has nothing to do with the scientific validity of the study, this practice can be justified on logistical and cost containment grounds. A trial that permits participants to present at any time of day or night to one of 50 sites, or any site of their choosing, would be dramatically more expensive and difficult to conduct than the same study conducted at five mandated sites on weekday mornings from 8 a.m. to noon. Even if a funder had the resources required to include large numbers of extraneous sites and staff them around the clock, paying those exorbitant costs would be inappropriate and wasteful.

While further examples could be considered, the point seems clear. The claim that conditions on trial participation are appropriate only when they are necessary, or at least useful for answering the scientific question or questions posed by the study is mistaken. And since scientific unity represents the most plausible substantive account of what a trial consists in, this argument rules out any attempt to explain the acceptability of conditions on enrollment in terms of their expression of the constitutive features of trial participation.

This conclusion raises the question of how else we can determine which conditions on enrollment in clinical trials are permissible, if not by reference to scientific necessity. The earlier, highly objectionable example of requiring participants to provide free car washing services to researchers illustrates that not every requirement is acceptable. But how, then, do we distinguish the appropriate from the inappropriate? The next section describes our preferred account.

## 2 APPROPRIATE CONDITIONS AS CONSISTENT WITH ETHICAL REQUIREMENTS

Why can it be acceptable to impose conditions that participants would prefer to refuse, even in studies that offer a prospect of clinical benefit, and even when the conditions are neither necessary nor useful to assessing the experimental treatment under study? The reason suggested by the forgoing examples is that clinical research is a complicated social institution that involves multiple stakeholders and has multiple goals, which give rise to multiple norms and ethical requirements. This, in turn, suggests that whether conditions for research participation are appropriate depends not just on whether they are scientifically necessary or useful, but on whether they help to satisfy the full range of ethical requirements on clinical trials.

Consider a brief analogy. Motorists must agree to a number of conditions when accessing public roads. They must agree to be licensed, registered, and insured. They must obey the speed limit, yield to emergency vehicles, and turn their headlights on at night. Many of these conditions are justified by the goal of promoting safety, which is presumably welcomed by most motorists. But conditions can also be justified by other legitimate goals which do not aim to protect the motorists themselves, for example, concerns about noise and air pollution, gasoline consumption, infrastructure costs, drains on police and medical services, and so on. For example, motorists can be required to have a muffler on their car, even when it increases costs and reduces speed.

Clinical trials are similarly governed by a range of legitimate goals and concerns, and while the interests and concerns of participants are critical, they are not all that counts. Provided a condition on research enrollment is justified by the broader range of relevant interests, and the corresponding ethical requirements they generate, the choice left to potential participants is to either agree to the condition or decline to enroll, much as the choice of a motorist is to either register and insure their car or to avoid driving on public roads.

Of course, the fact that research has multiple goals, and those goals give rise to multiple ethical requirements, does not on its own yield an understanding of which conditions on research participation are appropriate. We need to first know: what are the specific ethical requirements for clinical research? This is a matter of debate, and, when it comes to the present authors, we frequently disagree on the specifics. Nonetheless, there is a set of ethical requirements, which takes into account a broad range of interests, and the full set, whatever it might be, provides the standard against which to determine whether conditions on research enrollment are appropriate. Agreement on these claims is all that our present argument requires. Individuals who endorse different ethical requirements will disagree on whether some specific conditions are justified. Still, they will agree that appropriate conditions are not limited to those necessary or useful for answering the scientific questions posed by the study.

Granting that there is no consensus on the ethical requirements of clinical research, in what follows we will illustrate the implications of the present proposal by appealing to the widely‐endorsed view that there are seven ethical requirements for clinical research: social value, scientific validity, fair subject selection, favorable risk‐benefit ratio, independent review, informed consent, and respect for participants.^8^ Assuming, for illustrative purposes, that this is right, the present proposal amounts to the following claim: conditions on enrollment in clinical trials are appropriate if and only if they promote the satisfaction of these seven requirements.

Two of the requirements on this list—social value, and favorable risk‐benefit ratio—are endorsed by essentially every set of ethical requirements on clinical research of which we are aware. Why? Here is a standard answer: clinical research imposes burdens and harms on participants. These burdens and harms are, in themselves, a morally bad thing, but they are necessary for the generation of new medical knowledge. And the generation of new medical knowledge is extremely socially valuable. Moreover, greater social value offers stronger justification for the burdens and harms to which participants are exposed. The CIOMS guidelines contain an especially strong version of the social value requirement:

Researchers, sponsors, and research ethics committees must maximize the potential individual benefits of studies for both future patients and study participants. For instance, the social and scientific value of studies can be maximized by making data or specimens available for future research.^9^

This statement is strong because it requires not only that research has some social value, or that the amount it has meets some threshold of adequacy. Rather, it requires that social value be *maximized*. But we do not rely on a maximizing view. Instead, we will rely on just two claims about social value, which we will presently argue for: first, that social value should be *enhanced* where identified, feasible opportunities to do so exist (even if it is not maximized), and second, that the social value of a study can depend on, and include, the contribution it makes to the successful conduct of other, future valuable studies.

First, consider the requirement to enhance social value. To illustrate, imagine a study designed to rigorously evaluate a potentially valuable treatment in a way that satisfies all ethical requirements of clinical research. However, shortly before the study is initiated, a new method for administering the treatment under study is discovered which would require many fewer staff hours while not increasing the study costs or compromising the quality of the treatment or the study data in any way. By amending the protocol to use this new method instead of the old one, the researchers could greatly reduce the degree to which their study diverts clinic nurses who are needed to take care of patients in the community. Doing so would thereby enhance the study's social value by reducing the burdens its conduct imposes. In our view, absent compelling reason to the contrary, researchers who find themselves in that situation are required to change their plans to use the new method.

This requirement cannot be explained by claiming that, if researchers did not amend their design, their study would fail to produce enough social value to be justifiable in absolute terms. By description, the study was initially ethically designed when the option of using fewer nurses did not exist. Rather, the problem with failing to use the new method is that not doing so forgoes an identified, feasible opportunity to further enhance the study's social value. Even if the full‐on maximization of social value is not required because potentially too onerous, adopting easily available enhancements is.

Second, we now argue that the social value of one study can include its effects on other studies. We have already given one example: when researchers are choosing between a study design in which participants must visit five mandated sites, versus one where they have their pick of 50, the fact that the latter design would be much more expensive is an important reason to choose the former design instead. Note that this stewardship of funds does not directly improve any health outcome or yield any scientific knowledge. It is only indirectly, through being deployed in future research, that funds presently saved have any potential to generate social value. Yet researchers clearly have good reason to steward scarce funding resources, and the requirement to enhance social value through enabling future research best explains why that is so.

The notion that one study's social value can depend on its effects on others may seem like a departure from traditional research ethics; isn't the single study protocol, taken in isolation, the unit of evaluation for ethical oversight? While single studies may be the traditional unit of evaluation, that evaluation has always looked beyond any single study in order to include effects on future research. After all, it is a rare trial that has important social value independent of all other research. Particularly when it comes to early phase research, by nature phase 1 and 2 trials almost never generate clinically useful knowledge themselves. Rather, their point is that they enable phase 3 and 4 trials which do generate clinically useful knowledge. So if phase 1 and 2 trials were evaluated on their own, in isolation from the further research they enable, they would have inadequate social value and be uniformly unethical to conduct. But this is the wrong result. Instead, their social value ought to be understood in terms of the part they play in a development trajectory that includes other, future studies.

Indeed, as London and Kimmelman have recently argued, the very same study could be either valuable or not valuable at all depending on how well‐positioned to further future research it is. To use an example of theirs, a phase 2 trial is more valuable when it is one of a small number that is investigating promising indications which will predictably lead to phase 3 follow‐ups, but less valuable when it is one of many redundant phase 2 trials with no apparent prospects for further follow‐up.^10^ Even if they are otherwise identical, one trial is better‐positioned to enable valuable future research than the other and is for that reason itself more valuable. We hold that not only does the contribution studies make to future research matter when assessing their social value, but that contribution ought to be enhanced, just as other forms of social value ought to be enhanced. If minor adjustments to a phase 2 trial would yield valuable information for a range of planned phase 3 trials, the adjustments should be made.

Granted, in many realistic cases, researchers will have no feasible means for surveying a development trajectory or coordinating their trials with others. But it is important to remember that we have only endorsed a requirement to enhance social value where identifiable and feasible opportunities to do so exist. This is consistent with there also being many cases in which there are no such identifiable and feasible opportunities, and, of course, no one can be obligated to take opportunities that don't exist.

To summarize, we have claimed that conditions on clinical research participation are appropriate when they further the ethical values governing clinical research and illustrated that claim by assuming the common seven‐requirement framework. Among those requirements, we have emphasized a favorable risk‐benefit ratio and social value. With respect to social value, we have argued that feasible enhancements of social value are required and that social value itself must be understood broadly to include the effects a study may have on other, future studies. In our view, many conditions in clinical research that would appear mysterious or unjustifiable if scientific necessity were the only acceptable reason to impose a condition are instead obviously appropriate once one brings into focus the way that they improve the study's risk‐benefit ratio and/or enhance its social value.

Before moving on, there is a final complication to note. Measures that promote the satisfaction of one ethical requirement in clinical research can conflict with others. Including extra blood draws or scans can enhance social value but also increase participant risks. Explaining a study's purpose, and accurately describing its procedures enhances informed consent, but can undermine the scientific validity of studies that require deception. When a condition on enrollment furthers one ethical requirement while undermining another, it may be unclear whether, all things considered, the condition promotes the set of ethical requirements that apply to clinical research. While the question of how to resolve such conflict cases is extremely important, it is also a topic we will not address in any depth here. 11

Such conflict cases notwithstanding, when a condition on enrollment helps to satisfy one of those ethical requirements *without* conflicting with any of the others, it does clearly promote the set as a whole. Put positively, our analysis holds that contributing to the satisfaction of one or more ethical requirements while not being inconsistent with any of the others is sufficient for a condition to be ethically acceptable.

### 2.1 Voluntariness, coercion, and rights

Our account implies that conditions on research enrollment, including conditions participants would prefer to reject, may nonetheless be ethically appropriate or even required. Now suppose researchers are testing a novel intervention for a serious condition for which there are no currently existing therapies. Furthermore, suppose that a participant agrees to submit to several conditions on enrollment only because they are desperate and have no other options for receiving care. One might worry that this choice cannot reasonably be called voluntary. If we nonetheless allow that those conditions might be justified in light of, for example, the social value they produce, does that mean that our argument ultimately collapses into the claim that it is permissible to engage in the coercion of research participants, provided it's for a good cause?

In response to this kind of worry, we have already pointed out that essentially all voluntary agreements come with conditions, and we have given many examples in support of this point. But what we have not done is explained how this is possible. Philosophically speaking, why *aren't* those ordinary conditions we have pointed out coercive? And, more to the point, what guarantees that the conditions which our account supports aren't coercive? In this section, we answer these questions. We explain how choices made in unwelcome circumstances can still be voluntary and how the imposition of unwelcome conditions can correspondingly be non‐coercive. We also show that the conditions we recommend imposing are among those that are noncoercive even when they are unwelcome. To this end, we start with a brief note on the interrelated concepts of voluntariness, coercion, and rights.

There are several competing accounts in the literature of what constitutes voluntariness. Some emphasize a person's being free of controlling influences.^12^ Others focus on a person's possession of acceptable alternative choices.^13^ Against these proposals, though, consider a person with otherwise fatal cancer whose only treatment option is surgery. They may feel that they have no acceptable alternatives to consenting to the surgery, and they may experience little sense of personal control when making that decision. But those factors, although unfortunate, do not invalidate their consent to the surgery, nor do they make it wrong for the surgeon to perform it on them.^14^ This suggests that these accounts do not offer a means for assessing when the imposition of some condition on research enrollment is consistent with voluntariness, and when it isn't.

Instead, we understand voluntariness in the context of research in terms of freedom from coercion, and coercion as a kind of wrongful behavior. A prominent account of this type, due to Alan Wertheimer, holds that a person A coerces another person B when:

A threatens to deprive B of something

to which B is entitled as a matter of moral or legal right,

unless B does something for A.^15^

This account explains why a person with otherwise fatal cancer can nonetheless voluntarily consent to surgery. There is no (impossible) right to survive cancer without surgery, and in offering surgery the doctor is not threatening to deprive the patient of anything to which they have a right. Therefore, the doctor does not act coercively, and the patient can still render their voluntary consent to the proposed surgery, even though there is an important sense in which they have no choice but to accept. This account also captures the classic case of coercion in which a robber holds a gun to an individual's head and threatens to shoot unless the individual hands over their wallet. In this case, the robber *does* threaten to deprive the individual of something to which they have a right, that is, their personal safety. And as a result, the decision to surrender the wallet is a result of coercion, not voluntary consent.

To consider a case more analogous to enrolling in research that offers a chance of clinical benefit, suppose that my charity provides government‐funded dinners to indigent community members. Now suppose that I tell a community member who meets the kitchen's eligibility criteria that I won't let them eat unless they bring a bottle of wine. Given that they meet the kitchen's eligibility criteria, they have a right to access the program without my imposing further conditions. Hence I, like the robber, attempt to control their behavior by threatening to deprive them of something they are entitled to by right.

Now change the case. Imagine I tell an acquaintance who is in town for the week that I will cook them dinner, but only if they bring a bottle of wine. This scenario involves the same options: bring a bottle of wine and get dinner or get no dinner. It also involves my attempting to control or influence this person's behavior. I am, after all, trying to get them to bring a bottle of wine. But all the same, my offer is *not* coercive. The reason is that the acquaintance has no right to have dinner at my house at all. Hence, placing conditions on their ability to have dinner at my house does not threaten to deprive them of something to which they are entitled by right. These examples highlight a critical point: whether placing conditions on someone's access to a (potential) benefit is coercive depends on whether the person has a right to access it without the condition attached. What implications does this have for clinical research?

Potential participants do not have a right to demand that research be conducted in ways that contravene the valid principles of research ethics. For instance, participants cannot legitimately demand that studies they participate in select subjects unfairly, proceed absent independent review, or that they gratuitously avoid producing easily‐obtainable social value. Participants may sometimes want these things for all sorts of reasons, and even reasonably so, but they have no right to them. So once it is determined that a given condition on trial enrollment is all things considered appropriately related to the range of ethical values governing research, it follows that participants have no right to access the trial without accepting the condition. And *that*, on the analysis of coercion already outlined, means the decision to only offer trials with that feature is not coercive. This explains why the present proposal does not involve endorsing coercive conditions on research enrollment.

Having offered our account, and clarified its relationship to voluntariness and coercion, we are now in a position to take up the third and final task of our paper. To illustrate our view on appropriate conditions, the next section considers its implications for mandating that participants agree to permit their left‐over biosamples (e.g., saliva, blood, tissue) to be used for unspecified future research, including research unrelated to the study in question.

## 3 MANDATED USE OF BIOSPECIMENS FOR FUTURE UNSPECIFIED RESEARCH

Research on biological samples has enormous potential to contribute to improvements in health and wellbeing. As a result, the collection and storage of biological samples for future research has become nearly ubiquitous in clinical research. This practice has led to significant debate over what type of consent should be used for the collection and storage of biological samples. Because future studies may not be conducted for years, even decades, it is often not known at the time which studies will be conducted on the samples. Many commentators and guidelines thus endorse obtaining consent for a broad and unspecified range of future research, subject to a few limitations.^16^

Some studies make agreeing to the broad future use of left‐over samples optional, allowing individuals who refuse to still enroll in the study. This approach gives potential participants a choice, which they may appreciate, and likely has few downsides in cases where it is predictable that more than enough participants will agree. Data suggest that only a minority of individuals (4%–40%) would refuse to permit their left‐over samples to be used in unspecified future research;^17^ hence, all else held equal, when researchers need samples from only (say) 50% of participants, the optional approach should be sufficient. But, in other cases, it can be important for researchers to obtain samples from every, or almost every individual who participates in a trial. This raises a critical question: When the optional approach is likely to be insufficient, can it be acceptable for researchers to make agreeing to allow one's samples to be used for a broad range of future research a condition on enrollment?

As indicated already, OHRP has addressed this question in a determination letter and answered with a qualified “no.”^18^ For studies that offer participants a prospect of benefit, OHRP maintains that researchers *cannot* require them to permit left‐over biosamples to be stored and made available for unspecified future research. To comply with this ruling, the intramural NIH IRB now stipulates that protocols that offer the prospect of direct benefit must give participants the option of declining to have their samples used in future research. In contrast, this may be required for studies that offer “no prospect of direct benefit, for example, a repository or a study with only healthy volunteers.”^19^

OHRP defends their determination on the grounds that participants have a right to participate in the prospect of benefit research without having to contribute their biosamples for unspecified future research, hence, this requirement is coercive. In making this argument, OHRP agrees with our previous analysis of coercion and rights: the reason they claim that this requirement is coercive is that, in their view, participants have a right to access trials that offer the potential for clinical benefit without having to agree to the broad future use of their left‐over samples. But is there reason to believe participants have that right?

OHRP's determination letter emphasizes that the retention of left‐over biospecimens is for “unrelated” future research, as opposed to being part of the present study. This would violate participants' rights *if* they had a right against any conditions which are not necessary to the completion of the present study. But, this view is mistaken. As we have already seen, studies can impose conditions to enhance their social value, which includes supporting unrelated future research. This is why it is legitimate for researchers to place conditions on enrollment in clinical trials for the purpose of saving funds that can be devoted to future, unrelated research. It is also why early‐phase studies have genuine social value despite not generating clinically useful knowledge, and why early‐phase research ought to be designed in ways that maximize the likelihood of successful follow‐up trials.

By contrast, in our approach, whether the requirement to donate left‐over biosamples is appropriate depends on how it related to the full set of ethical requirements for clinical research. If this requirement furthers some ethical requirements, while frustrating none, it follows that it does not violate participants' rights. We now argue that this is frequently the case: a requirement to donate left‐over samples provides a feasible opportunity to enhance the social value of many studies, while not frustrating any other ethical requirement.

We have already explained why, given the potential for samples to promote future health and wellbeing, mandating their storage for future use will frequently enhance studies' social value. Much like saving money, saving samples frees up resources for the conduct of valuable future research and is therefore presumptively desirable from the perspective of enhancing social value. But this still leaves the question of whether this requirement conflicts with any of the other ethical requirements in the common seven‐requirement framework we have accepted for the sake of argument and illustration. Among those, there is no reason to suspect such a mandate conflicts with the requirements for scientific validity or independent review. That leaves the potential for conflict with the requirements for a favorable risk‐benefit profile, respect for participants, informed consent, and fair subject selection. The next three sections consider whether mandating the collection and storage of biospecimens for unspecified future research necessarily conflicts with any of these requirements.

### 3.1 Inconsistent with a favorable risk‐benefit profile?

Even if requiring the collection and storage of samples for unspecified future research produces social value, it could still fall afoul of the requirement for a favorable risk‐benefit ratio if the social value it stands to produce is too scant in relation to the personal risks it imposes on participants, or if the personal risks are too high in absolute terms. However, the incremental risks to participants from their left‐over samples being used for future, unspecified research are extremely low. Indeed, the absence of reports of moderate or serious harm in the hundreds of millions of individuals who have had biospecimens collected for research, suggests that, so long as appropriate protections are in place, this use poses almost no risks to participants.^20^ Hence, it is highly implausible that this requirement will be made unacceptable by virtue of violating the constraint on acceptable participant risk.^21^

### 3.2 Inconsistent with informed consent?

One might think the requirement to donate left‐over biosamples is inconsistent with informed consent just because it is a requirement, and all requirements are coercive and therefore inconsistent with obtaining voluntary consent. We have already explained why this is not the case. Is there any other reason to think that this condition is incompatible with obtaining valid consent?

One might hold that there is a conflict here because the biosamples are being stored for *unspecified* future research. If future research is unspecified, how can participants' consent to surrender the biosamples be sufficiently informed? For instance: what if, unbeknownst to the participants, the specific future research in which their samples will be used includes methods or purposes the participants would find disturbing or would rather reject?

In fact, most uses of biospecimens in research are innocuous, which is part of why even voluntary donation rates are high. Still, there are some uses of biosamples that are sensitive. For example, some individuals have objections to their samples being used for research to develop methods to clone human beings. Such possibilities provide an objection against storing participants' biospecimens for future research without any limitations whatsoever using a blanket consent. But such possibilities can also be anticipated, and participants can be informed of potentially charged uses and given the opportunity to opt‐out of them. The storage and future use of biological samples are widely regarded as appropriate when it includes such safeguards.^22^

Furthermore, if the inherent uncertainty surrounding the future uses of stored samples rendered their collection inherently inconsistent with obtaining informed consent, this would be true not just when samples are left‐over from the evaluation of interventions that offer a prospect of direct benefit. It would be true in wholly non‐beneficial research whose entire point is to collect and store biospecimens for future research. The fact that many studies which obtain informed consent exist for just that purpose, and are widely regarded as acceptable, undermines this view.

### 3.3 Inconsistent with respect for research subjects and/or fair subject selection?

One might worry that placing conditions on research participation that are not necessary or even useful for achieving the scientific goal(s) of a study is inconsistent with the ethical requirement to treat research subjects with respect. If researchers can mandate the storage of biospecimens for unspecified future research, including research that is not related to the present study, what is to prevent them from mandating any conditions that they like? Can they mandate that research participants contribute to the researcher's favorite charity? Presumably, that would be inappropriate. But why? After all, raising money for charity produces social value.

To assess this concern, first, suppose a requirement that participants contribute to researchers' favorite charities were institutionalized: suppose, for instance, that the NIH were to launch an initiative encouraging all funded research to include such a requirement. That policy would be baffling. Requiring research participants to pay would conflict with fair subject selection if it is assumed, as it commonly is, that fairness requires members of different socioeconomic classes to have equal access to the benefits of research participation. It would also undermine the social value of research, by skewing the participant population toward those of greater means. Our view need not countenance such a policy, and it is anyway farfetched.

By contrast, suppose, somewhat more plausibly, that a single researcher were to attempt to include such a requirement in their protocol. This researcher would still face the same objections relating to fair subject selection and scientific validity. But they would also face another one. Given that there is no adequately institutionalized understanding of donations to charity as within the scope of research participation, any researcher who unilaterally acted in this manner would be acting outside of their sanctioned role, qua researcher, and would instead be treating the participants of whom they made this demand as a source of resources for their private ends. Even if those private ends are charitable ones, it is plausible that this behavior would inherently violate the requirement to treat participants with respect. Requiring subjects to contribute left‐over biosamples, by contrast, does not have these features. It does not undermine the scientific validity of studies or unfairly exclude the poor. It does not involve researchers acting outside of their authority to further their private aims. Rather, it is another instance of researchers generating social value by systematically collecting data that can be used to protect and promote health and well‐being.

A different way that requiring participants to donate left‐over samples might disrespect them or conflict with a fair selection of subjects would be by disregarding potential participants' centrally held value commitments. For example, the Yanomami culture has practices with respect to the treatment of remains that conflict with the retention of left‐over biospecimens.^23^ These cultural practices raise important concerns about how researchers ought to respect participants' beliefs and what forms of accommodation are appropriate—issues similar to those raised in other areas of research and care by Jehovah's Witnesses' rejection of blood products, or some cultural and religious attitudes on acceptable organ use. Yet, as this parallel indicates, the existence of these belief communities does not entail that mandates to allow the use of samples for future research are illegitimate in *all* studies, any more than the existence of Jehovah's Witnesses entails the illegitimacy of mandates to accept blood products in all studies (e.g., studies that include surgeries where odds of survival without blood products is very low).

Unless it is necessary to obtain samples from all participants, researchers should be willing to make exceptions in these exceptional cases. But, when researchers cannot selectively relax this requirement and still achieve their aims, their situation calls for more detailed analysis. Because the view we have taken up here does not hierarchically rank the ethical requirements of clinical research, there is no general solution to cases of genuine conflict between social value and fair subject selection. Regardless, the important point is that the existence of such hard cases does not suggest that it is inappropriate to mandate the future use of left‐over samples when doing so does not conflict with any of the other ethical requirements of clinical research.

In our view, most cases are easy cases—mandating the donation and storage of biosamples for unspecified future research adds minimal personal risks while enhancing social value. Because researchers ought to enhance social value whenever possible, they ought to require participants to agree to the collection and storage of their biosamples for unspecified future research in many cases. If this is correct, not only is imposing this condition on participation generally appropriate, it is frequently ethically required.

## 4 CONCLUSION

To what extent is it permissible for investigators to place conditions on individuals' participation in clinical trials, especially trials which offer a prospect of clinical benefit? Individuals do not have a right to participate in trials that offer a prospect of clinical benefit without any conditions at all. At the same time, investigators may not place whatever conditions they want on research participation. What, then, makes some conditions appropriate and others not?

In this paper, we began by rejecting one answer to this question. According to that answer, conditions are acceptable only when they express constitutive features of a trial; researchers are only permitted to impose conditions that are either necessary or at least extremely useful to the scientific success of the trial. We then offered our own answer: research aims at multiple goals and is subject to multiple ethical requirements, and conditions on participation are justifiable when they promote the full set of those requirements. We then showed how, on an appropriate understanding of rights, voluntariness, and coercion, conditions which are justified in that way may be unwelcome, but they are not coercive. We concluded with a case study of the requirement that participants agree to donate left‐over biosamples for unspecified future research. We found that there is good reason to believe this requirement will frequently be justified in terms of its ability to enhance studies' social value while not conflicting with any other ethical requirements. This case study both shows how our view can fruitfully be applied and supports a change to U.S. regulatory policy, which currently forbids a practice that should be more broadly instituted, not discouraged or prohibited.

## CONFLICT OF INTEREST

The authors declare no conflict of interest.

## Footnotes

1 Jansen, L. A., & and Wall, S. (2018). Reconsidering paternalism in clinical research. *Bioethics*, *32*(1), 50–58. Note that we are not here discussing the extent to which patients might have a right to try experimental interventions outside of a clinical trial. FDA. (2020). *Right to try*. http://www.fda.gov/patients/learn-about-expanded-access-and-other-treatment-options/right-try

2 Wertheimer, A. (2011). *Rethinking the ethics of clinical research* (p. 140). Oxford University Press.

3 OHRP. (2012). *Re: Human research subject protections under Federalwide Assurance (FWA)—4969*. http://wayback.archive-it.org/4657/20150826185216; http://www.hhs.gov/ohrp/detrm_letrs/YR12/aug12a.pdf. We discuss the reasoning in this letter in section 3.

4 In the wake of the death of Ellen Roche in a phase one trial at Johns Hopkins, an OHRP investigation led to the suspension of 2400 of its trials, which had enrolled over 15,000 participants, and the freezing of $300 million in funding. PBS. (2001). *Research halt*. https://www.pbs.org/newshour/show/research-halt. Such action is very rare, but still a useful reminder that OHRP holds legal authority over most human subjects research.

5 In the literature, Douglas MacKay has defended a view in this vicinity. Specifically, he offers a view of fair subject selection on which justice requires that researchers only distinguish among potential participants in the ways necessary to accomplishing their goals *qua* researchers, which are primarily (and perhaps solely) to answer scientific questions. MacKay, D. (2016). Fair subject selection in clinical research: Formal equality of opportunity. *Journal of Medical Ethics*, *42*(10), 672–677. More recently, MacKay and Saylor offer a more involved but recognizably similar analysis of fair subject selection. A theme in both pieces is that when enrolling potential participants, researchers should not decide between them on the grounds of minimizing their personal risk (e.g., by enrolling ones without comorbidities). However, it is important to note that MacKay and Saylor confine their analysis to specifying what fairness consists in, while still allowing that “considerations of fairness are not the only legitimate considerations relevant to participant selection.” MacKay, D., & Saylor, K. (2020). Four faces of fair subject selection. *The American Journal of Bioethics*, *20*(2), 5–19, p. 17. Because their analysis is limited to specifying what fairness consists in, and because it allows that fairness is not the only legitimate consideration, their analysis does not answer the present question of what makes a condition on research participation appropriate all things considered.

6 This defense is implicit in OHRP's determination letter, as we explain in Section 3. It was also explicitly raised by an anonymous reviewer.

7 While we are not aware of any explicit articulation and defense of the scientific necessity view in the literature, as noted above, related ideas have been developed by MacKay and MacKay and Saylor, op. cit. note 5, and we also believe this view is implicit both OHRP's determination as well as comments from several reviewers.

8 Emanuel, E. J., Wendler, D., & Grady, C. (2000). What makes clinical research ethical? *Journal of the American Medical Association*, *283*(20), 2701–2711.

9 Council for International Organizations of Medical Sciences. (2016*). International ethical guidelines for health‐related research involving humans* (p. 11). https://cioms.ch/wp-content/uploads/2017/01/WEB-CIOMS-EthicalGuidelines.pdf

10 London, A. J., & Kimmelman, J. (2019) Clinical trial portfolios: A critical oversight in human research ethics, drug regulation, and policy. *Hastings Center Report*, *49*(4), 31–41, p.34. London and Kimmelman argue for the claim that there must be some new form of ethical oversight that can operate at the research portfolio level. Here, we just note that even when evaluation is undertaken at the study level, it can still include foreseeable effects on other parts of a portfolio.

11 On the seminal principlist approach to bioethics, when moral principles conflict, that conflict should be resolved by balancing the relevant concerns—a process which must be applied case‐by‐case and which cannot be spelled out mechanically. If this is correct, then there is not much interesting or informative to say about how to resolve conflict cases in purely general terms. Beauchamp, T., & Childress, J. (2012). *Principles of biomedical ethics* (7th ed.). Oxford University Press.

12 Nelson, R. M., Beauchamp, T., Miller, V. A., Reynolds, W., Ittenbach, R. F., & Luce, M. F. (2011). The concept of voluntary consent. *The American Journal of Bioethics*, *11*(8), 6–16.

13 Olsaretti, S. (1998). Freedom, force and choice: Against the rights‐based definition of voluntariness. *Journal of Political Philosophy*, *6*(1), 53–78.

14 Wertheimer, A. (2012). Voluntary consent: Why a value‐neutral concept won't work. *The Journal of Medicine and Philosophy*, *37*(3), 226–54.

15 Additionally, B must have no reasonable alternative but to comply. This further condition will not be relevant, however. Wertheimer, A. (2014). *Coercion*. Princeton University Press.

16 This approach is often referred to as “broad consent.” However, this term is now used in the revised research regulations to describe a specific approach to consent, with a number of stipulations. To avoid confusion, we will thus not use the term broad consent. For readers who think in those terms, we are essentially referring to what is sometimes called “old” broad consent, as opposed to “new” broad consent. Grady, C., Eckstein, L., Berkman, B., Brock, D., Cook‐Deegan, R., Fullerton, S. M., Greely, H., Hansson, M. G., Hull, S., Kim, S., Lo, B., Pentz, R., Rodriguez, L., Weil, C., Wilfond, B. S., & Wendler, D. (2015). Broad consent for research with biological samples: Workshop conclusions. *The American Journal of Bioethics*, *15*(9), 34–42.

17 Ibid: 5.

18 OHRP, op. cit. note 3.

19 OHRP. (2022). *Consent template for use at the NIH Clinical Center*. https://ohsrp.nih.gov/confluence/display/ohsrp/Consent+Templates+and+Guidance

20 Wendler, D. & Rid, A. (2015). Genetic research on biospecimens poses minimal risk. *Trends in Genetics*, *31*(1), 11–15.

21 The only possible exceptions would be cases, gestured at above, in which the researchers are confident that optional provision will yield a fully scientifically adequate set of samples; this because it is wrong to impose even minimal risks gratuitously.

22 Grady, C., et al., op. cit. note 16.

23 Survival International. (2015). *Brazil: Blood samples returned to Yanomami after nearly 50 years*. https://www.survivalinternational.org/news/10727

